# Surface Flame-Retardant Systems of Rigid Polyurethane Foams: An Overview

**DOI:** 10.3390/ma16072728

**Published:** 2023-03-29

**Authors:** Yuping Jiang, Hongyu Yang, Xiang Lin, Simeng Xiang, Xiaming Feng, Chaojun Wan

**Affiliations:** College of Materials Science and Engineering, Chongqing University, Chongqing 400044, China

**Keywords:** rigid polyurethane foam, flame retardant, surface coating, fire safety

## Abstract

Rigid polyurethane foam (RPUF) is one of the best thermal insulation materials available, but its flammability makes it a potential fire hazard. Due to its porous nature, the large specific surface area is the key factor for easy ignition and rapid fires spread when exposed to heat sources. The burning process of RPUF mainly takes place on the surface. Therefore, if a flame-retardant coating can be formed on the surface of RPUF, it can effectively reduce or stop the flame propagation on the surface of RPUF, further improving the fire safety. Compared with the bulk flame retardant of RPUF, the flame-retardant coating on its surface has a higher efficiency in improving fire safety. This paper aims to review the preparations, properties, and working mechanisms of RPUF surface flame-retardant systems. Flame-retardant coatings are divided into non-intumescent flame-retardant coatings (NIFRCs) and intumescent flame-retardant coatings (IFRCs), depending on whether the flame-retardant coating expands when heated. After discussion, the development trends for surface flame-retardant systems are considered to be high-performance, biological, biomimetic, multifunctional flame-retardant coatings.

## 1. Introduction

RPUF is one of the best options for building insulation materials due to its advantages such as low density, low thermal conductivity, high specific strength, easy production, and low cost [1]. On the other hand, due to its porous structure and high content of aliphatic segments, RPUF is highly flammable and burns violently in the air, emitting toxic gases and smoke after ignition, which severely affects human life and property [2,3]. Therefore, it is all important and necessary to improve the flame retardancy and fire safety of RPUF. These have been studied for years, and generally by constructing bulk flame-retardant RPUF systems (BFR/RPUF) by physically or chemically incorporating flame retardants (additive-type or reactive-type) into the RPUF matrix [4].

The incorporation of flame retardants into the RPUF matrix by physical blending prior to foaming is one of the earliest flame-retardant methods. The polymer chain structure of RPUF is unaltered and the process is simple. Among the additive types, halogenated flame retardants have been studied and used earlier, but they are being phased out due to adverse environmental effects. Research into improving the flame retardancy of RPUFs with aluminum hydroxide [5,6,7], expandable graphite (EG) [8,9], ammonium polyphosphate (APP) [10,11,12], and melamine (MEL) [13,14,15] is relatively mature. In addition, synthetic organic flame retardants containing phosphorus are rapidly developing due to their environmental friendliness, high flame-retardant efficiency, ease of modification, etc. [16,17,18,19,20]. Zhang et al. [16] synthesized a flame retardant from 9,10-dihydro-9-oxa-10-phosphaphenanthrene-10-oxide (DOPO), benzaldehyde, and aniline; the limit oxygen index (LOI) of RPUF increased to 28.1% and the char residue increased from 6.1% to 15.3% in the cone calorimeter test.

The additive-type flame retardants have some problems in the RPUF system, such as poor dispersion, compatibility, and interfacial adhesion, resulting in the unsatisfactory flame-retardant efficiency and short lifetime [21]. More durable flame retardancy can be achieved by incorporating modified diols or polyols containing phosphorus and/or nitrogen into the polyurethane (PU) structure through chemical bonding [22,23]. Yuan et al. [24] synthesized two polyols, BHPP (P-containing) and MADP (N-containing), which partially replaced polyester polyols in EG-containing RPUF systems. As a result, the thermal stability and flame retardancy were effectively promoted, the LOI value increased to 33.5%, the peak heat release rate (p-HRR) decreased from 188 kW/m^2^ to 136 kW/m^2^, and the total heat release rate (THR) decreased by 34% when 70% polyester was equally replaced by BHPP and MADP.

The BFR/RPUF enhances the flame retardancy of the RPUF and even makes it self-extinguishing. In this case, the surface foams burn while the internal foams remain, indicating that the flame retardancy of the BFR/RPUF is mainly achieved by consuming the surface foams with flame retardants to form a char layer (in the condensed phase). The remaining large proportion of the internal flame retardant is not effective, so the flame-retardant efficiency of BFR/RPUF is low [4,21].

The surface flame-retardant RPUF system (SFR/RPUF) is constructed by applying a flame-retardant coating to the surface of the combustible substrate, which has a higher flame-retardant efficiency than the BFR/RPUF and would not degrade the excellent physical properties of the RPUF. The research and application of flame-retardant coatings on flexible polyurethane foam and other polymeric materials are more extensive [25,26]. However, due to the closed-cell structure of RPUF, the coatings need to have a higher flame-retardant efficiency or greater thickness to achieve a significant flame-retardant level, which is the main problem of SFR/RPUF. In recent years, many scientists have devoted themselves to the research of SFR/RPUF and have made great progress. In this paper, we review the progress and give a brief outlook.

## 2. Non-Intumescent Flame-Retardant Coating

Fire-retardant coatings, common materials for fire protection of buildings, are widely used in the construction industry. Because they do not change the nature of the matrix, while greatly improving the ability of the matrix to resist flame propagation, they significantly reduce the fire hazard of buildings [27,28]. Flame-retardant coatings are divided into NIFRC [29,30,31,32] and IFRC [33,34,35], depending on whether the flame-retardant coating expands when heated. NIFRCs are generally composed of inorganic materials that can prevent the return of the heat and oxygen supply to the combustible matrix during the heating process of the polymer and, at the same time, prevent the transfer of combustible gas generated by the pyrolysis of the matrix to the flame, thus protecting the polymer matrix [4]. According to the literature reports, NIFRCs for RPUF mainly include hydrogel/sol [29,30], aerogel [32], ceramic materials [36], etc.

### 2.1. Hydrogel and Silica Sol

Hydrogel is a substance with a 3D reticular structure, usually consisting of cross-linked tangles of hydrophilic polymers. Hydrogel coatings contain large amounts of water, are environmentally friendly, and are increasingly being used in flame-retardant applications [37]. Hydrogels, such as montmorillonite/xylose hydrogel [38], di-(triethoxysilylpropyl) phenylphosphamide hydrogel [39], maize straw-co-2-acrylamide-2-methylpropanesulfonic acid-co-acrylic acid hydrogel [40], poly (N-isopropylacrylamide) (PNIPAAm)/sodium alginate (SA)/silver nanoparticles (AgNPs) thermosensitive network hydrogel [41], PNIPAAm/SA/polyvinyl alcohol hydrogel [42], PNIPAAm/SA [43], konjac glucomannan (KGM)/fly ash (FA) hydrogel [44], and SA/sodium carboxymethylcellulose (CMC)/N-isopropyl acrylamide (PNIPAM) hydrogel [45] are prepared and effectively improve the fire safety of various substrates.

**Figure 1:**
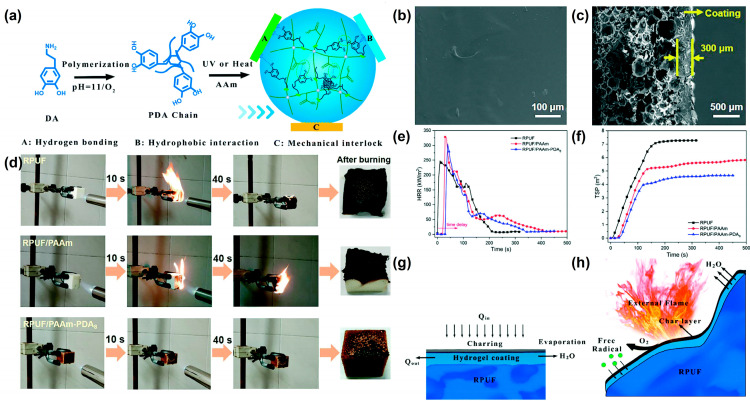
(**a**) Schematic diagram of the synthesis route of the PAAm–PDA hydrogel, (**b**) SEM image of the surface of the hydrogel-coated RPUF, (**c**) SEM image of the cross-sectional structure of the hydrogel-coated RPUF, (**d**) video screenshot of the burning process of uncoated and hydrogel-coated RPUF under the propane flame for 10 s, (**e**) HRR and (**f**) TSP curves of uncoated and hydrogel-coated RPUF, (**g**) and (**h**) are schematic of the flame-retardant mechanisms of the hydrogel-coated RPUF [29].

Jiang et al. [29] introduced polydopamine (PDA) chains into polyacrylic-polydopamine (PAAm) hydrogels to form PAAm-PDA double network hydrogels to improve the toughness and the adhesion strength of the hydrogel coating. The schematic diagram of the synthesis route of PAAm-PDA coating is shown in Figure 1a; an ultraviolet (UV) curing lamp or 60 °C oven is used to cure the precursor solution and form the hydrogel coating on the RPUF surface. The surface of the hydrogel-coated RPUF is uniform and smooth (Figure 1b). The cross-section structure of the hydrogel-coated RPUF shows that the dense hydrogel coating is attached to the porous surface of the RPUF (Figure 1c). Due to the introduction of PDA, the adhesion strength of PAAm-PDA hydrogel to RPUF increases significantly from 67.5 kPa to 191.25 kPa, compared to the PAAm hydrogel. After coating with the PAAm-PDA hydrogel, RPUF shows an excellent self-extinguishing performance (Figure 1d), the time to ignition (TTI) increases from 6 s to 36 s, the heat release rate (HRR) decreases after reaching the peak values (Figure 1e), and the total smoke production (TSP) decreases by 42.2% (Figure 1f) in the cone test. As shown in Figure 1g, the hydrogel coating releases a lot of water vapor when it is attacked by heat. Therefore, the mechanisms of the improvement of the PAAm-PDA coating for RPUF are speculated as (i) the heat absorption and cooling effect of water during evaporation after heating, (ii) the presence of hydrogel coating delaying the ignition of foam, (iii) the char layer that forms after carbonization of the hydrogel playing a protective role, and (iv) water vapor diluting the combustible gas and oxygen concentration (Figure 1h).

**Figure 2:**
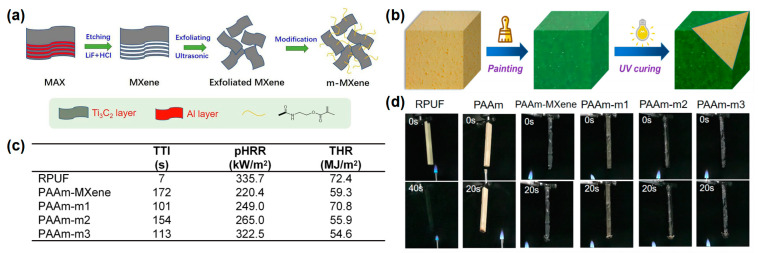
(**a**) Schematic diagram of preparation route of m-MXene nanosheets; (**b**) UV curing process of coated RPUF, (**c**) TTI, pHRR, and THR data of samples in cone test; (**d**) video screenshot of pure RPUF and coated RPUF samples during UL-94 testing [30].

Yang et al. [30] reported an organic-inorganic hydrogel modified with MXene nanosheets for RPUF coating. To improve the dispersion of MXene nanosheets in the hydrogel, MXene is first modified by grafting with double bonds, and then introduced into an polyacrylamide (PAAm) hydrogel by radical polymerization to prepare MXene-based hydrogel coating (PAAm-MXene) (Figure 2a,b). The RPUFs coated with PAAm hydrogels, appended with 10 mg of MXene and 5 mg, 10 mg, and 15 mg of modified MXene, were named as PAAm-MXene, PAAm-m1, PAAm-m2, and PAAm-m3, respectively. From the cone result, the TTI was significantly delayed after coating with the PAAm-MXene hydrogel. Compared to the pure RPUF, the p-HRR and THR of PAAm-m2 decreased from 335.7 kW/m^2^ and 72.4 MJ/m^2^ to 265 kW/m^2^ and 55.9 MJ/m^2^, respectively (Figure 2c). In the UL-94 test, the pure RPUF was easily ignited, and the flame quickly burned to the top of the specimen, while the coated RPUF could not be ignited after 2 10-s of flame applications (Figure 2d). The above results showed that the construction of the hydrogel coating on the RPUF surface made the RPUF more difficult to ignite, prolonging the ignition time, providing time for the personnel to escape, and in fact significantly reducing the fire hazard of the RPUF.

**Figure 3:**
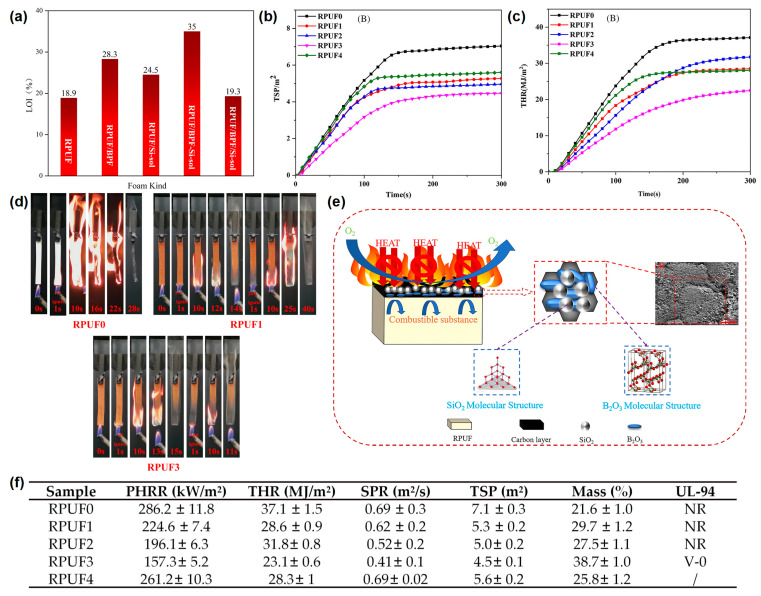
(**a**) The histogram of LOI values of pure RPUF and treated RPUF samples, (**b**) THR, and (**c**) TSP curves of pure RPUF and treated RPUF samples; (**d**) video screenshot of pure RPUF, RPUF coated with BPR, and RPUF coated with BPR/Si-sol during UL-94 test; (**e**) schematic diagram of the flame-retardant mechanism of treated RPUF samples; (**f**) the data of p-HRR, THR, SPR, TSP, residual mass in cone test, and UL-94 test of pure RPUF and treated RPUF samples [31].

In addition to hydrogels, silica sol (Si-sol) has also been used to prepare flame-retardant coatings for RPUF. Xu and Zhong [31] reported a boron phenolic resin (BPR)/Si-sol coated RPUF with a high flame-retardant efficiency. From the LOI test results, the BPR/Si-sol hybrid coating reached a 35 vol % and was higher than those of RPUF coated with BPR (28.3 vol %) or Si-sol (24.5 vol %), indicating that the synergistic effect exists between BPR and Si-sol exists during the combustion process (Figure 3a). The RPUFs coated with different BPR and/or Si-sol were designated as RPUF 0–4 in the cone test and the UL-94 test. The peak smoke production rate (p-SPR) and TSP of the RPUF coated with BPR were significantly reduced compared to that of the pure RPUF, indicating that the presence of BPR could effectively inhibit the smoke production in the process of combustion. For the sample of RPUF coated with BPR and Si-sol, the p-SPR and THR were further reduced compared to that of the RPUF coated with BPR, indicating the synergistic effect between BPR and Si-sol on the smoke suppression of RPUF (Figure 3b,f). The p-HRR and THR of the RPUFs coated with BPR/Si-sol hybrid coating decreased by 45% (from 286.2 kW/m^2^ to 157.3 kW/m^2^) and 37.7% (from 37.1 MJ/m^2^ to 23.1 MJ/m^2^), respectively, compared to the pure RPUF (Figure 3c,f). From the video screenshot during the UL-94 test in Figure 3d, the pure RPUF was easily ignited as shown in other reports. The sample of RPUF coated with BPR could not be ignited in the first 10 s flame application but burned violently after the second 10 s flame applications, indicating that this sample could not pass any rating in the UL-94 test. When BPR was combined with Si-sol, the sample could quickly self-extinguish after both of the 2 10 s flame applications and could pass the V-0 rating in the UL-94 test, indicating that the BPR/Si-sol hybrid coating could observably prevent flame propagation along the surface of the RPUF. The flame-retardant mechanisms are speculated as follows: (i) BPR can form a char layer on the surface of the RPUF due to the rich benzene ring structure and (ii) the Si-O-Si bond formed from the Si-sol can further improve the high-temperature ablation resistance of the char layer (Figure 3e).

### 2.2. Aerogel

The aqueous gels can be converted into aerogels using advanced drying technology, replacing the internal liquid with gas. The aerogel consists of a microporous solid and is made from inorganic compounds or organic polymers [32]. Among them, silica aerogels, an ideal candidate for both thermal insulation and flame-retardant applications, have been extensively studied [32,46,47,48,49,50]. The unique nanoporous network skeleton of the aerogel confers special properties, such as low density, low thermal conductivity, and small average pore diameter. Therefore, many aerogels have been used as coatings to improve the flame retardancy of organic matrices [22,51,52,53].

**Figure 4:**
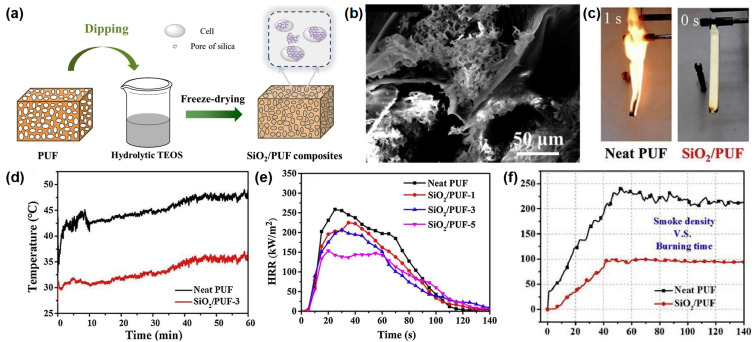
(**a**) Preparation route of SiO_2_/RPUF composites, (**b**) SEM image of SiO_2_/RPUF, (**c**) digital images of pure and SiO_2_ aerogel-coated RPUF, (**d**) temperature vs. time curves of the upper surface points in thermal conductivity test, (**e**) HRR in cone test of pure PUF and SiO_2_ aerogel-coated RPUF, and (**f**) specific density of neat and SiO_2_ aerogel-coated RPUF in smoke chamber test [32].

Zhao et al. [32] reported a novel method to prepare silica aerogel on the surface of RPUF. The results show that it significantly improves the flame retardancy and the smoke suppression of RPUF while maintaining the inherent properties. The SiO_2_/PUF composites are prepared by a series of processes, as shown in Figure 4a. The neat RPUF with specific dimensions is dipped in a hydrolysate of tetraethyl orthosilicate (TEOS); during the dipping process, the TEOS can attach to the bubble wall of the RPUF and form a gel in situ. After ageing, the samples are soaked and washed with water and then freeze-dried to obtain the final products. After the freeze-drying process, the silica aerogel with nanopores is formed on the surface of the RPUF with macropores (Figure 4b). The SiO_2_/PUFs with 1 mL, 3 mL, and 5 mL TEOS are named as SiO_2_/PUF-1, SiO_2_/PUF-2, and SiO_2_/PUF-3, respectively. In the UL-94 test, pure RPUF is easily ignited with no rating, while the SiO_2_/PUF passes the UL-94 V-0 rating and self-extinguishes immediately after ignition, demonstrating its excellent fire safety (Figure 4c). Infrared thermal imaging tests are carried out to illustrate the good thermal insulation of SiO_2_ aerogel-coated RPUF. Remarkably, the temperatures of the upper surface points of SiO_2_/PUF-3 are always much lower than those of pure RPUF for the same heating time, indicating the better thermal insulation property of SiO_2_/PUF composites (Figure 4d). From the cone test, the pure RPUF has the highest p-HRR value (260 kW/m^2^), while the p-HRR value of the SiO_2_ aerogel coated RPUF gradually decreases with the increase in the SiO_2_ aerogel amount, which means that the effective protective barrier effect of silica aerogels can significantly retard the combustion of RPUF (Figure 4e). From the smoke density curve in Figure 4f, the specific optical density of SiO_2_/PUF is reduced by 55.7%, which may be due to the barrier effect of the SiO_2_ aerogel and the SiO_2_ aerogel does not produce smoke when heated. The flame-retardant mechanism can be speculated as follows: a SiO_2_-rich hybrid char layer forms during the combustion process and acts as a barrier to protect the RPUF matrix from further combustion.

**Figure 5:**
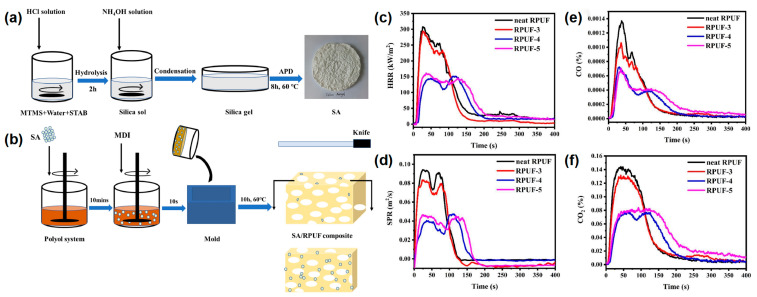
(**a**) The schematic diagram of the SA manufacturing process; (**b**) the preparation route of SA-coated RPUF, (**c**) HRR, (**d**) SPR, (**e**) CO, and (**f**) CO_2_ curves in the cone test of pure RPUF and SA-coated RPUF [47].

Yan et al. [47] also reported a type of castor oil-based RPUF coated with silica aerogel by the sol-gel method of methyltrimethoxysilane (MTMS). In their work, the silica aerogel was successfully prepared by the sol-gel method of MTMS under the atmospheric drying process, as shown in Figure 5a. Two types of silica aerogel/SA/RPUF composites were prepared by adding SA during the foaming process of RPUF and by coating SA on the RPUF surface, respectively (Figure 5b). The RPUF with 0 wt% and 15 wt% SA powder were named as neat RPUF and RPUF-3. The dipped and dried neat RPUF and RPUF-3 were named as RPUF-4 and RPUF-5, respectively. From the cone test, the first and second p-HRR values of RPUF-4 were reduced by 52.43% and 40.78%, respectively, compared to the pure RPUF (Figure 5c). The first and second p-SPR were reduced by 56.86% and 47.82%, respectively. The RPUF-4 and RPUF-5 showed a remarkable decrease in the first and second p-SRP values compared to those of the neat RPUF and RPUF-3 (Figure 5d). The average CO production of RPUF-3, RPUF-4, and RPUF-5 decreased from 0.0029 kg/kg of pure RPUF to 2.3 g/kg, 1.5 g/kg, and 1.7 g/kg, respectively. (Figure 5e). The average CO_2_ production of RPUF-3, RPUF-4, and RPUF-5 decreased from 0.27 kg/kg of pure RPUF to 0.25 kg/kg, 0.16 kg/kg, and 0.19 kg/kg, respectively (Figure 5f). From the above analysis, it can be seen that the formation of a silica aerogel layer on the surface of RPUF significantly improved the fire safety of RPUF and had a great application potential for improving the fire safety of the building insulation materials.

**Figure 6:**
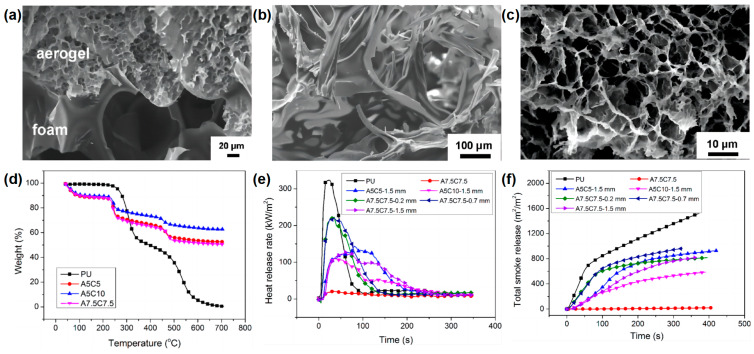
SEM micrograph of (**a**) RPUF/aerogel interface; Char residue of (**b**) RPUF and (**c**) aerogel after LOI test; (**d**) TGA curves of pure RPUF and alginate/clay aerogels with different ratios; (**e**) HRR, and (**f**) TSR curves of samples in cone test [50].

In addition to the silica aerogel, organic-inorganic aerogel has also been reported as a flame-retardant coating for RPUF. Chen and co-workers [50] reported the flame-retardant application of the alginate/clay aerogel coating on the surface of RPUF by the freeze-drying method. In the preparation route of PU foam coated with alginate/clay aerogel coatings, the alginate/clay suspensions were coated on the surface of RPUF and then frozen and dried using a freeze-dryer. The alginate/clay aerogel with different thicknesses and different alginate and clay contents can be formed on the surface of the RPUF. Figure 6a shows that the pore sizes of the aerogel and the RPUF were very different. The pore sizes of the RPUF closed cells were several hundred microns, while the pore sizes of the aerogel were 10–30 μm. The thermal stability of the RPUF matrix and the alginate/clay aerogel coating were very different (Figure 6d). Pure RPUF is a highly flammable material due to its LOI value of only 17 vol %, whereas the alginate/clay aerogel A7.5C7.5 can self-extinguish even in a pure oxygen atmosphere. With the increasing of the coating thickness and clay content, the LOI of the RPUF coated with alginate/clay aerogel gradually increased. The sample of RPUF coated with 1.5 mm of A7.5C7.5 could self-extinguish even in a pure oxygen atmosphere, indicating that the aerogel provides an excellent protection against the flame. From the SEM micrograph of the char residue of RPUF and aerogel (Figure 6b,c), it can be seen that the shape of the char residue of the RPUF shrinks, while the shape of the aerogel remains after burning in the LOI test. The unaltered aerogel shell protects the RPUF from further combustion, resulting in self-extinguishing even in an atmosphere with a very high oxygen concentration. From the cone results, the alginate/clay aerogel-coated RPUF significantly reduced the p-HRR and TSR compared to those of pure RPUF (Figure 6e,f), indicating that the alginate/clay aerogel coating had a very good fireproofing effect on RPUF. The above results indicate that this simple and inexpensive method has important practical significance for imparting higher fire safety to RPUF and has a potential application value.

### 2.3. Ceramic

The flame-retardant mechanism can be either in the vapor phase or in the condensed phase. In the condensed phase, flame retardants accelerate the degradation of the polymer and form an insulating dense char layer on the polymer surface [54]. The dense layers reduce the heat conduction, block the entry of oxygen, and reduce the concentration of combustible gas and the generation of consistent toxic smoke, thus protecting the RPUF [55].

**Figure 7:**
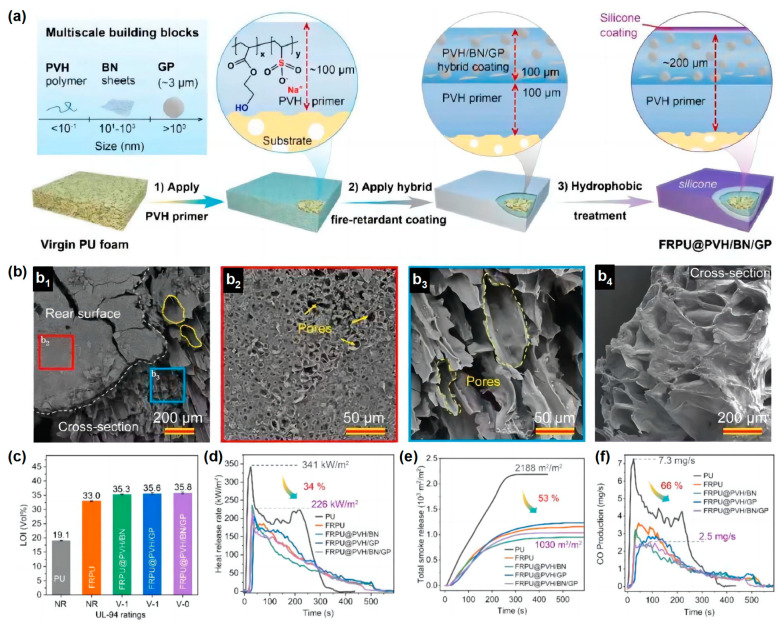
(**a**) The scheme of the manufacturing process of FRPU@PVH/BN/GP; (**b**) porous structure of char layer; (**c**) LOI value of neat RPUF and coated RPUF; (**d**) HRR, (**e**) TSR, and (**f**) CO curves in cone calorimetry test [36].

Similarly, inspired by the flame resistant, high-temperature flowable and low thermal conductivity properties of volcanic lava, a ceramizable multi-scale flame-retardant coating material has been developed by Song and co-workers to provide effective fire protection for many building materials [36]. They prepared a multi-scale organic/inorganic hybrid PVH/BN/GP flame-retardant coating material using aqueous copolymer poly (hydroxyethyl acrylate sodium vinyl sulfonate) (PVH) as the coating matrix, introducing a low melting point glass powder (GP) as the ceramic precursor and boron nitride (BN) as the synergist. PVH (~100 μm) and PVH/BN/GP (~100 μm) were deposited on the substrate surface (Figure 7a). The coating itself is highly flame retardant and can provide ideal fire protection for a wide range of base materials. At temperatures above 350 °C, the GP particles in the hybrid coating softened and eventually melted completely at high temperatures (>650 °C) to form a flowing melt. The GP melt can be used as a high-temperature adhesive to fill in the macroscopic cracks on the surface of the organic char layer, eventually forming a dense and complete ceramic protective layer (Figure 7b). In addition, the thermal degradation of the organic PVH coating substrate can promote the formation of pores in the char layer, and its porous structure is very similar to the structure of volcanic lava, giving the char layer a thermal conductivity as low as 0.0897 W/m·K at 700 °C. The RPUF treated with ~200 μm coating showed a maximum LOI of 35.8 vol % and achieved the ideal V-0 rating in the UL-94 test. The total smoke release (TSR) and peak CO production (PCOP) were significantly reduced by 53% and 66%, respectively, while the compressive strength significantly increased by 41% (Figure 7c–f). The overall performance of the material was superior to that of RPUF reported in the previous literature. This work provided a novel strategy for the production of an economical flame-retardant coating for the combustible substrate where fire protection is required.

## 3. Intumescent Flame-Retardant Coating

The IFR coatings expand when heated to prevent the RPUF from burning. Most conventional intumescent flame retardants (IFRs) consist of three components: an acid source (dehydrating), carbon source (carbon forming), and gas source (foaming agent) [56]. The intumescent flame-retardant coating can be prepared by adding conventional IFRs (such as EG) to the coating substrate [33,34] or by reacting the flame retardant with the coating substrate (all-in-one) [57,58].

### 3.1. Conventional Flame-Retardant in Coating

The addition of EG can significantly improve the flame-retardant properties of the matrix. Due to the decomposition of the intercalated EG compounds and the oxidation of acids, a large number of gases (such as carbon dioxide, water, and sulphur dioxide) are produced during the heating process. The gases accumulate between the EG layers to form a large air pressure, causing the distances between the EG layers to increase rapidly, forming an efficient oxygen insulation structure.

**Figure 8:**
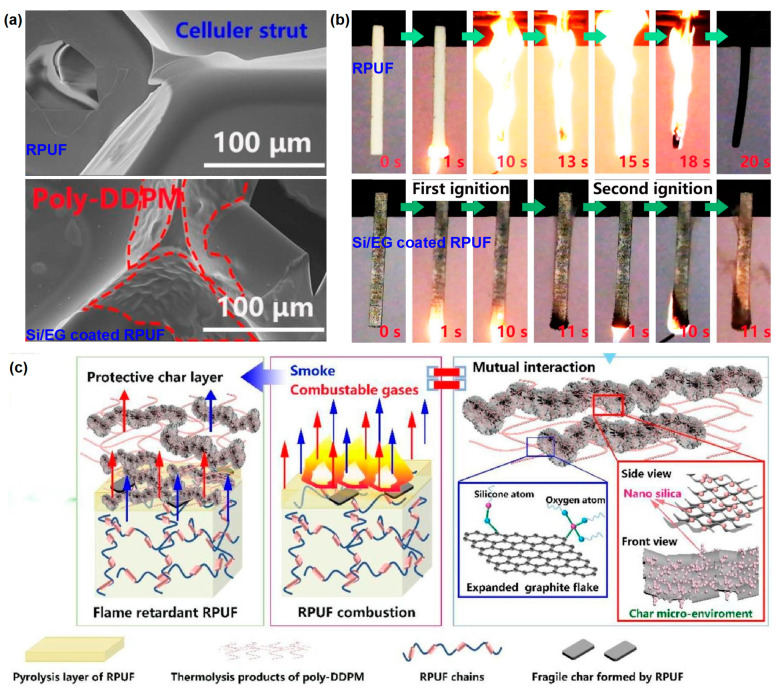
(**a**) Surface SEM images of pure RPUF and the coated RPUF samples, (**b**) optical photos of samples in UL-94 testing, and (**c**) flame-retardant mechanism schematic diagram of the RPUF with Si/EG coating [33].

Wang et al. [33] synthesized an organosilicon oligomer (DDPM) and mixed the DDPM with EG and brushed it onto the RPUF surface. Figure 8a shows the surface SEM images of the RPUF samples. The polyhedral-shaped cavities of RPUF and RPUF with Si/EG coating are almost identical. The Si/EG coated samples have folded cell walls due to the cured poly-DDPM. The flame retardancy characterizations (LOI, UL-94, and cone test) show that the Si/EG coating significantly increases the flame resistance of the RPUF samples. Compared to pure RPUF, the LOI value of RPUF with Si/EG coating increased from 18 vol % to 32.3 vol % and passed the V-0 rating in the UL-94 test (Figure 8b); the p-HRR and the peak smoke release rate (p-SRR) were reduced by 55% and 59%, respectively. The potential flame-retardant mechanism is speculated as being the following (Figure 8c): during the heating process, due to the release of gases such as SO_2_ and NO_2_, EG expands rapidly, and the graphite flakes form more cracked holes. These spaces provide micro-sites for the mutual interaction of EG with the thermal cleavage products of poly-DDPM. The pyrolysis products of RPUF with Si/EG coating promote the formation of carbon residual. Such a carbon layer has a good strength. It is well insulated from oxygen, protects the substrate, and prevents heat transfer. In addition, the compressive strength of the Si/EG-coated RPUF samples is improved.

**Figure 9:**
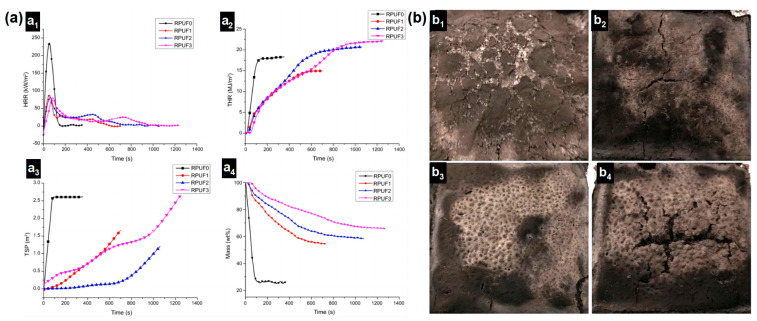
(**a**) Cone results of pure RPUF and coated RPUF ((**a_1_**). HRR curves; (**a_2_**). THR curves; (**a_3_**). TSP curves; (**a_4_**). Mass loss curves); (**b**) digital photos of the char residue after cone tests ((**b_1_**). RPUF0, (**b_2_**). RPUF1, (**b_3_**). RPUF2, (**b_4_**). RPUF3) [34].

A waterborne intumescent fire-retardant coating is an environmentally friendly coating due to the use of water as a solvent. It is a relatively common fire-retardant coating and is widely used in the field of the fire protection of steel structures. Yang [34] prepared a fire-retardant coating for RPUF and investigated the flame retardancy and combustion performance of pure RPUF and coated RPUF. In their work, a type of waterborne intumescent fire-retardant coating was prepared using the classical P-N-C intumescent fire-retardant system, which includes APP, PER, MEL, silicone acrylic emulsion, other additives, and water. The specimens were prepared by evenly applying the waterborne intumescent fireproof coating to the RPUF surface. Specimens of RPUF with different coating thicknesses (0, 0.25, 0.5, and 1 mm) were prepared and abbreviated as RPUF 0–3. From the cone results, it can be seen that the presence of waterborne intumescent fire protection coating on the surface of RPUF markedly increased the TTI; reduced the p-HRR, THR, and TSP of RPUF; and notably reduced the mass loss during combustion, indicating that the waterborne intumescent coating could significantly improve the fire safety of RPUF (Figure 9a). From the photographs of the char residue after the cone tests, as shown in Figure 9b, it can be observed that the char residues were more compact and stronger with the coatings. From the fire protection mechanism of the coated RPUF, the fire protective coating on RPUF could act as a physical barrier for RPUF to resist fire. Therefore, the application of the waterborne intumescent fire-retardant coating on the surface of RPUF is also an effective and economical strategy to improve the fire safety of RPUF.

### 3.2. All-in-One

As IFRs are compounded with various components, there are problems with ratio control, poor thermal stability, and uneven phase distribution. To overcome these problems, the concept of an “all-in-one” IFR has been proposed: an intumescent flame retardant that integrates three sources into one macromolecule [59].

“All-in-one” IFR has a good flame resistance, stable thermal performance, low smoke, low toxicity, and good compatibility with polymer materials, which are important development trends in the field of IFR. Miao and co-workers [35] used poly(dimethoxy)-phosphazene (PDMP) elastomer as an all-in-one IFR to prepare a cotton fabric coating based on the one-step impregnation method. In the VFT process, the cotton treated with 5.3% PDMP exhibited self-extinguishing properties. After 50 wash cycles, the samples still had a high LOI value and retained self-extinguishing properties. The PDMP-coated cotton exhibited significant and efficient flame-retardant properties. Ding [60] prepared a new microencapsulated APP (MAPP) with APP as the core and cross-linked β-cyclodextrin (HDI-CD) as the shell through a strong chemical bond between the shell layers. When applied to polypropylene (PP), MAPP showed a good compatibility and dispersion. The PP/MAPP23 composites achieved a V-0 rating in the UL-94 test with an LOI value of 30.3%. The results of TG-FTIR-MS showed that MAPP modified the thermal degradation of PP and reduced the generation of combustible fragments. With the action of MAPP degradation products, the carbon residue was formed and became stronger. L Zhang and co-workers [61] successfully prepared a novel integrated flame-retardant polyelectrolyte complex (PAPP) using poly diallyldimethylammonium chloride (PDDA) and APP as the raw materials. PAPP provides an effective flame resistance for PP. Pan et al. [59] prepared an all-in-one IFR by ion-exchange reaction between APP and piperazine sulfonate. This IFR could impart a stronger flame resistance, good smoke inhibition, and better mechanical properties to PP.

**Figure 10:**
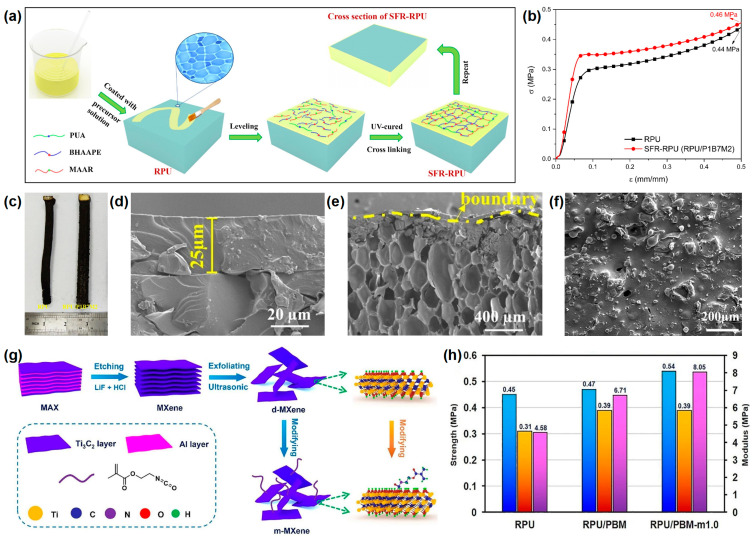
(**a**) Illustration of the SFR-RPU foam fabrication, (**b**) compression strength curves of pure RPU and RPU/P1B7M2, (**c**) photographs of pure RPU and RPU/P1B7M2 after UL-94 testing, (**d**) SEM of the interface where the RPU foam contacts with the coating, (**e**) cross-section and (**f**) surface of RPU/P1B7M2 in UL-94 test [57], (**g**) fabrication route of m-MXene nanosheets, (**h**) mechanical bar graphs of RPU, RPU/PBM, and RPU/PBM-m1.0 [58].

The application of all-in-one IFRs in RPUF has also received some attention. Huang’s group investigated a novel nitrogen-phosphorus-containing UV-curable self-extinguishing coating. It was compounded with the surface of RPUF to obtain an SFR-RPUF. [57] The fabrication process of SFR-RPUF is shown in Figure 10a. The compressive strength of SFR-RPUF is higher than that of pure RPUF, indicating improved compressive properties (Figure 10b). The SFR-RPUF system has a self-extinguishing effect when a 25-μm thick coating was introduced (Figure 10d). During combustion, the SFR-RPUF coating transformed into a dense and smooth self-extinguishing expanding carbon residue, preventing further combustion of the underlying foam and reducing the flame spread (Figure 10c,e,f). In addition, the group combined the advantages of organic IFR and MXene nanosheets (a 2D nanomaterial) to produce an IFR/MXene nanocomposite coating. It was sprayed on the surface of RPU as a coating with flame retardant and UV-curable effects. [58] The fabrication process of the surface-modified MXene nanosheets is shown in Figure 10g. Compared to the pure RPUF, the compressive strength, yield strength, and modulus of the coated RPUF at 50% strain were significantly improved (Figure 10h). RPU/PBM-m1.0 also exhibited better mechanical properties compared to RPU/PBM, which was attributed to the co-crosslinking between m-MXene and the IFR coating, resulting in a good dispersion and less aggregation of the flakes.

### 3.3. Bio-Based

Many IFRs are mainly derived from non-renewable fossil fuels and are not sustainable [62]. With the development of society, people have put forward higher requirements for environmentally friendly, safe, and efficient IFRs. Research on the production of IFRs from biomass materials as raw materials has received wide attention due to their green, renewable, and degradable characteristics [44,63,64].

**Figure 11:**
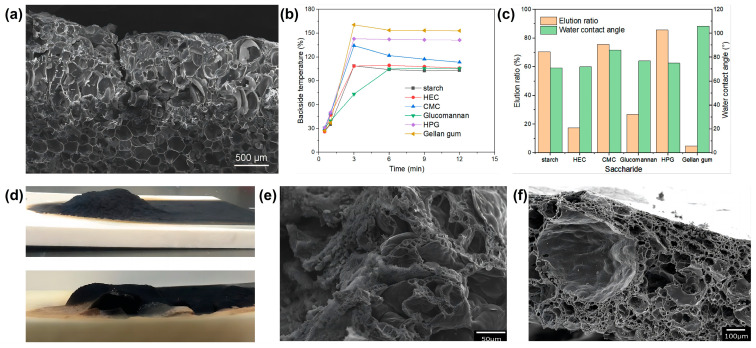
(**a**) SEM micrograph of the cross-sectional SPB/starch mixture coated RPUF after heating for 12 min in the pre-mixed flame. (**b**) Backside temperature versus time curves of the different coatings. (**c**) Water contact angles and elution ratios of the coatings (SPB mixed with different saccharides) on the surface of RPUF [65]. (**d**) The appearance of the coated RPUF (**top**: SPB/HEC; **bottom**: SPB/gellan gum) after sustaining 12 min of heating in the pre-mixed flame. (**e**) SEM micrograph of the cross-sectional carbon residue of SPB/HEC mixture-coated RPUF. (**f**) SEM micrograph of the cross-sectional carbon residue of SPB/gellan gum mixture-coated RPUF [66].

In 2011, Tsuyumoto and co-workers [65] used the char-forming property of starch and the non-combustibility of amorphous sodium polyborate (SPB) to mix starch with SPB as flame retardants and applied the mixtures to the RPUF surface (Figure 11a). The RPUF with a 10-mm thick mixture showed high flame-retardant properties. It withstood the 12-min exposure to the 100 mm fire of the premixed gas burner without igniting or burning. When the coated RPUF was exposed to the fire, the starch reacted as an IFR, simultaneously promoting the generation of foam and carbon residue. Starch improved the adhesion of SPB to RPUF, but the coating (starch mixed with SPB) was sensitive to moisture (Figure 11c).

Subsequently, many efforts have been made by them to prepare a variety of polysaccharides/SPB coatings. Many types of saccharides are selected for use as flame retardants and adhesion promoters, such as hydroxyethyl cellulose (HEC), gellan gum, etc. [66]. These coatings (different saccharide/SPB composites) have good flame-retardant properties. Compared to SPB/starch, the flame resistance of the coatings (mixed with HEC, carboxymethyl cellulose, 2-hydroxypropyl guar gum, glucomannan, and gellan gum) is better due to the lower binding. The vertical burner test (pre-mixed butane burner heated with a 100 mm flame) shows that the coated RPUF (10-mm thick) can withstand ignition for 12 min with its back side maintaining a temperature in the range of 100 °C to 160 °C (Figure 11b). All of these RPUFs with different saccharide/SPB coatings carbonize and swell to 5–15 mm like IFR (Figure 11d). It is easy to see in the SEM images (Figure 11e,f) that SPB foams develop on the surface of the coated RPUFs. The presence of the SPB foam results in the formation of a thick carbon residue layer. Both of them protect the interior from heat and O_2_. The saccharides selected later have a higher adhesion and crystallinity than the SPB/starch mixture, which could effectively prevent the flame spread and improve flame resistance.

**Figure 12:**
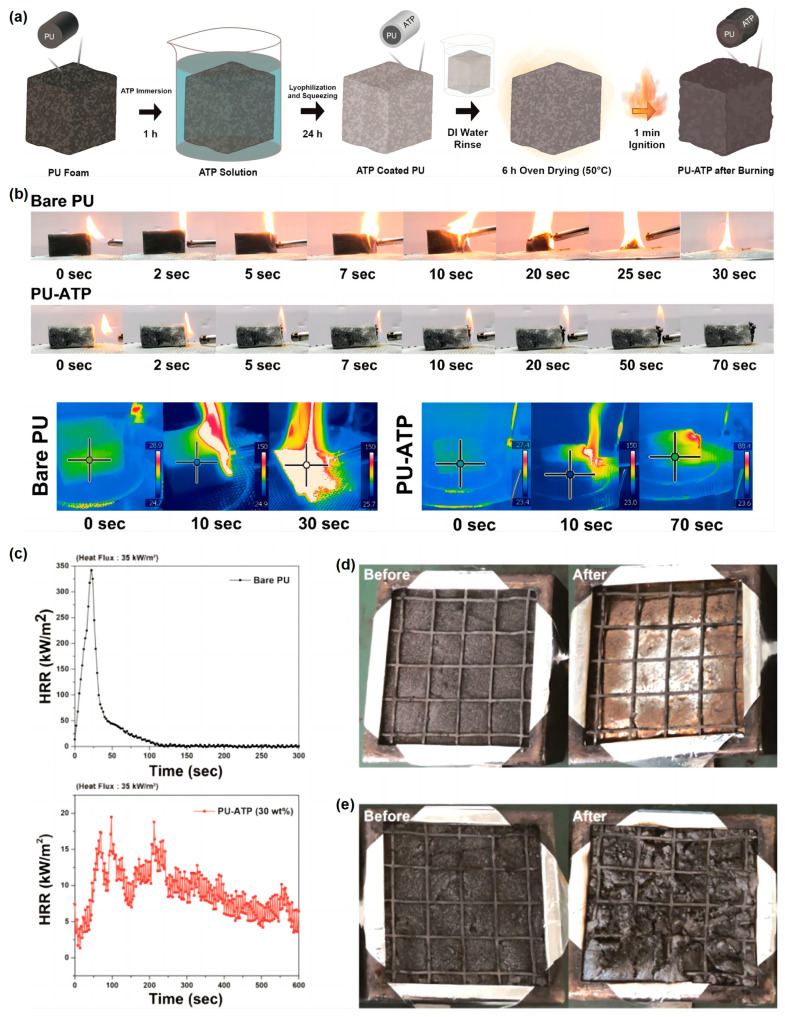
(**a**) The schematic diagram of the manufacturing process of PU-ATP. (**b**) Digital images of pure PU and PU-ATP (with 30% load) during the direct burning test. (**c**) HRR of pure PU and PU-ATP (with 30% load). Digital images before and after the cone test of (**d**) pure PU and (**e**) PU-ATP (with 30% load) [67].

Bio-based materials often contain polyhydroxy groups, which have been more widely studied as carbon sources. There are relatively few acid sources in bio-based materials and in recent years there have been studies using adenosine triphosphate (ATP) as a flame retardant. Jeong et al. [67] found that the application of ATP to polyurethane foam (PU-ATP) by a simple dipping process can significantly improve its fire safety. The PU-ATP foams are prepared, as shown in Figure 12a. Direct burning experiments showed that the PU-ATP foam almost completely failed to ignite when exposed to a torch flame (Figure 12b). Compared to pure PU, PU-ATP (with 30% load) demonstrated a longer ignition time and a reduction in p-HRR (94.3%) (Figure 12c). Importantly, ATP exhibited a tremendous increase in volume, whereas the volume increase in the typical phosphorus-containing FR was minimal (Figure 12d,e).

## 4. Modification of SFR/RPUF

**Figure 13:**
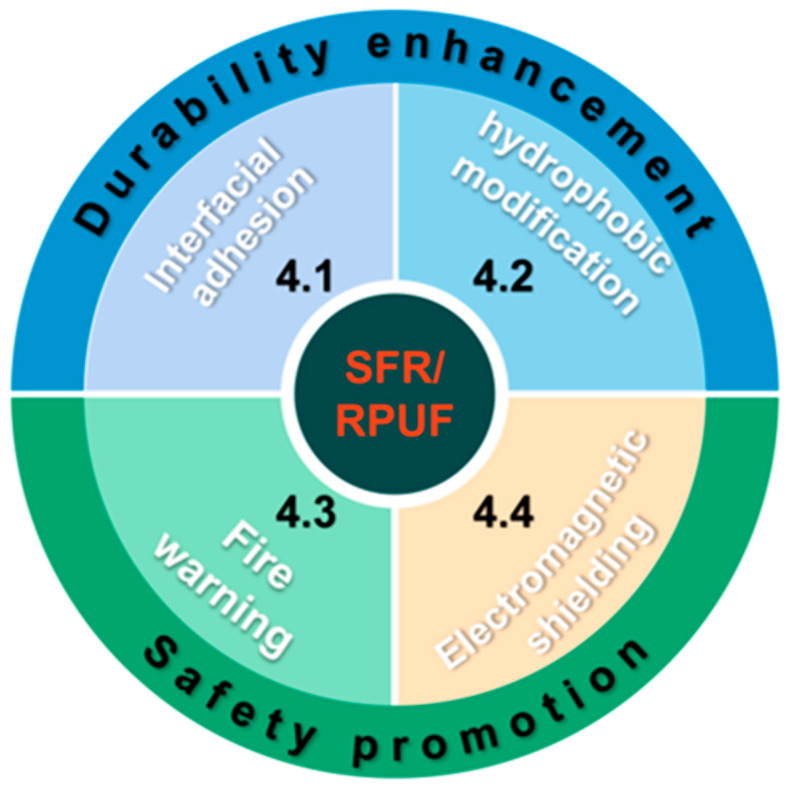
Illustration of the SFR/RPUF modification.

In general, flame-retardant coatings can provide good fire protection performances for RPUF and improve fire safety. However, as living standards improve, high performance SFR/RPUF with a good durability, fire warning, or electromagnetic shielding is needed to meet the increasingly stringent quality standards and safety requirements. This section aims to introduce the functional modification of SFR/RPUF, which is divided into two parts, durability enhancement and safety promotion (as shown in Figure 13).

### 4.1. Interfacial Adhesion Property

The interfacial adhesion between the RPUF and the coating and the hydrophobic property of the coating surface are critical to the durability of the SFR/RPUF. Due to the weak interfacial adhesion, the flame-retardant coating easily falls off the RPUF surface during storage and use, which limits its practical application, so it is necessary to improve the interfacial adhesion and hydrophobic properties of the flame-retardant coating [68].

**Figure 14:**
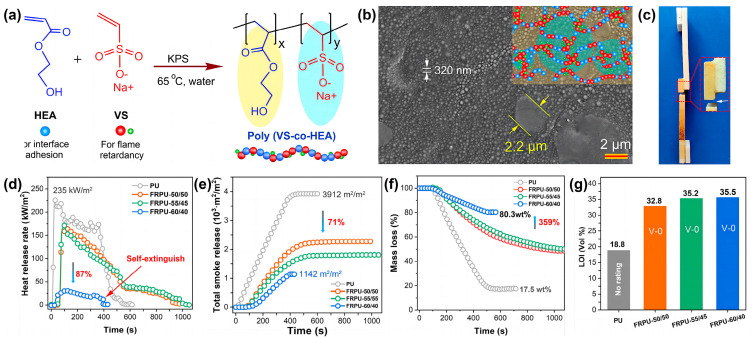
(**a**) Schematic diagram of the synthetic route of poly (VS-co-HEA); (**b**) the phase-separated micro/nanostructure of poly (VS-co-HEA); (**c**) digital photo of poly (VS-co-HEA) coatings versus PU foam following shear testing, during which bulk PU foam broke before interfaces; (**d**) HRR, (**e**) TSR, and (**f**) mass loss curves in UL-94 test; (**g**) LOI of RPUF [69].

Referring to the adhesion mechanism of snails and tree frogs in the biological world, Ma Zhewen et al. [69] synthesized an advanced waterborne polymer coating (VS-co-HEA) with high viscosity and flame retardancy by simple free radical copolymerization using hydroxyethyl acrylate (HEA) and sodium vinyl sulfonate (VS) as the raw materials. The copolymer contains an abundance of hydroxyl groups, and a large number of H bonds enhance the interfacial adhesion. In addition, the sodium sulfonate group in VS improves the flame retardancy of the coating, and also induces a phase-separated micro/nanostructure, resulting in mechanical interlocking with the rough PU surface and further improving the adhesion. They take advantage of interfacial hydrogen bonding and mechanical interlocking to create a fire-retardant polymeric nanocoating with phase-separated micro/nanostructures by radical copolymerization. The synthesis process of poly (VS-co-HEA) is illustrated in Figure 14a. In order to balance the fire retardancy and adhesion, three predesigned copolymers with VS/HEA ratios of 50/50, 55/45, and 60/40 were prepared and coated on the surface of RPUF, designated as RPUF-50/50, RPUF-55/45, and RPUF-60/40, respectively. The typical phase-separated micro/nanostructure of poly (VS-co-HEA) is shown in Figure 14b. Due to the interfacial hydrogen bonding and the interfacial linkage with the substrate, there is strong adhesion with RPUF, and the shear strength is improved to 2.2 MPa. The strong interfacial adhesion is present between the synthetic aqueous coating and RPUF (Figure 14c). In the cone calorimeter test, the p-HRR is reduced by 87% (Figure 14d) and the TSR is reduced by 71% compared to the pure RPUF sample (Figure 14e). Poly (VS-co-HEA)/RPUF shows a good self-extinguishing performance after the coating thickness reaches 600 μm (Figure 14d). The UL-94 rating is V-0 and the LOI reaches 35.5 vol % (Figure 14g).

### 4.2. Hydrophobic Modification

In order to achieve a good long-term environmental stability in practical applications, some researchers have sought to improve the hydrophobicity of samples [69,70,71]. The further hydrophobic treatment reduces the wet sensitivity of the coating and shows superhydrophobic characteristics.

**Figure 15:**
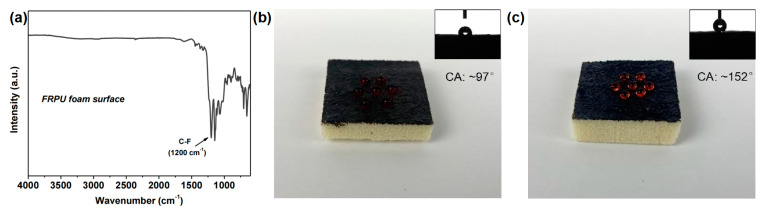
(**a**) IR spectra of hybrid flame-retardant coating on FRPU foam surface. Due to surface hydrophobic treatment, a strong peak at ~1200 cm^−1^ can be observed, which is assigned to C-F; Digital and corresponding contact angles photos (inset) of FRPU samples (**b**) before and (**c**) after hydrophobic treatment [72].

Ma et al. [72] designed and synthesized a water-soluble polyamino molecule called HCPA by modifying hexachlorophosphazene (HCCP). They then fabricated GO/HCPA hybrid networks from HCPA and graphene oxide sheets. The GO/HCPA had excellent mechanical flexibility, exceptional flame retardancy and excellent expansion effect; it could be used to effectively cover flammable RPF with flame-retardant nanomaterial. In addition, they also used HFTS to hydrophobically modify the surface. Figure 15a shows the infrared spectrum of the composite flame-retardant coating on the RPUF surface. Due to the hydrophobic treatment of the surface, a strong peak at ~1200 cm^−1^ can be seen, which is attributed to C-F. In Figure 15b,c corresponding contact angle photographs of FRPU samples before and after hydrophobic treatment. These results indicate that the modified SFR/RPUF has good hydrophobic properties.

**Figure 16:**
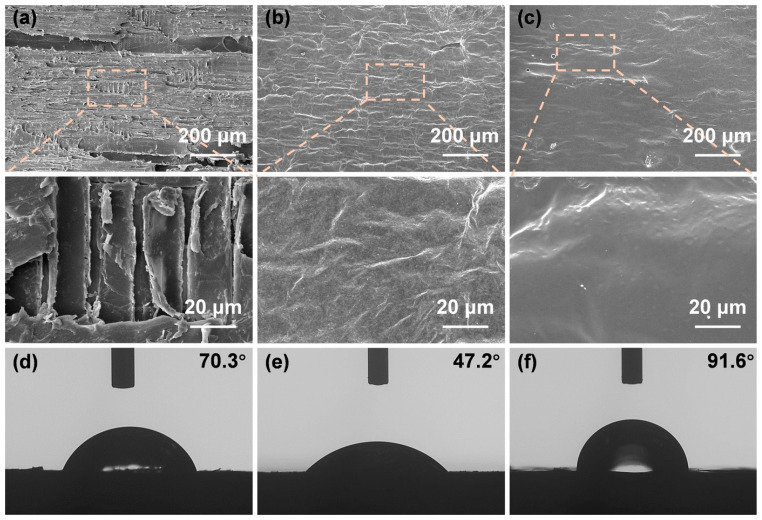
SEM images of (**a**) the untreated wood, (**b**) MXene-coated wood, and (**c**) waterborne acrylic resin (WA) on MXene-coated wood. Water contact angle after 1 s of (**d**) natural wood, (**e**) MXene-coated wood, and (**f**) waterborne acrylic resin (WA) on MXene-coated wood [73].

Wei et al. [73] improved the environmental stability of MXene-coated wood by coating it with water-based acrylic resin (WA). The microstructures of untreated wood, MXene/wood, and WA-MXene/wood were examined by SEM. Cavities and cracks were clearly visible on the surface of the untreated wood. Because certain small cracks were filled or covered with MXene sheets, MXene/wood had a much smoother surface compared to the rough surface of the untreated wood (Figure 16a,b). Further deposition of the WA coating resulted in a smoother WA-MXene/wood surface (Figure 16c) and increased the hydrophobicity with a contact angle of approximately 91.6° (Figure 16f).

### 4.3. Fire Warning

The fire spreads quickly and people take a long time to react, causing serious economic loss and even loss of life. Using temperature-sensitive materials to detect high temperature heat sources and fire-retardant coatings to provide a fire warning function, people’s response times to fire hazard can be reduced, thus reducing the fire risk of combustible materials. Materials such as GO and MXene, whose resistance varies by several orders of magnitude under high temperature conditions, can be used as fire alarm sensors [72,74].

**Figure 17:**
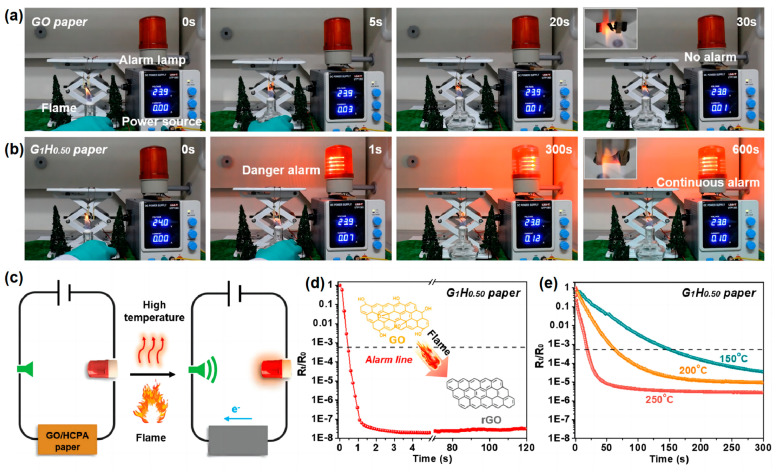
Images of (**a**) GO and (**b**) G_1_H_0.50_ paper’s flame detection methods. (**c**) A schematic representation of the fire alarm sensor based on GO/HCPA paper operating in a high-temperature or flame attack scenario. Electrical resistance transition behavior of the G_1_H_0.50_ paper under (**d**) flame attacks and (**e**) different ambient temperatures [72].

Cao et al. [72] used a multi-amino molecule called HCPA, which can be used to decorate graphene oxide (GO) sheets and create GO/HCPA hybrid networks while performing the triple functions of crosslinking, flame retardant, and reducing agent. The design and operation of the GO/HCPA hybrid network-based fire early warning system is shown in Figure 17c. The GO sheet was thermally reduced and formed a conductive path when the flame or high-temperature conditions were reached, activating the warning lamp. Figure 17a,b shows the relevant flame detection and early warning processes. The resistance transition curve of G_1_H_0.50_ in the paper under flame erosion conditions is shown in Figure 17d. The high-temperature warning response behavior and corresponding resistance change of G_1_H_0.50_ are also performed and observed on paper under various high-temperature conditions, As shown in Figure 17e. GO/HCPA exhibits an ultra-long alarm period (>600 s) and an ultra-fast fire alarm response time (~0.6 s).

**Figure 18:**
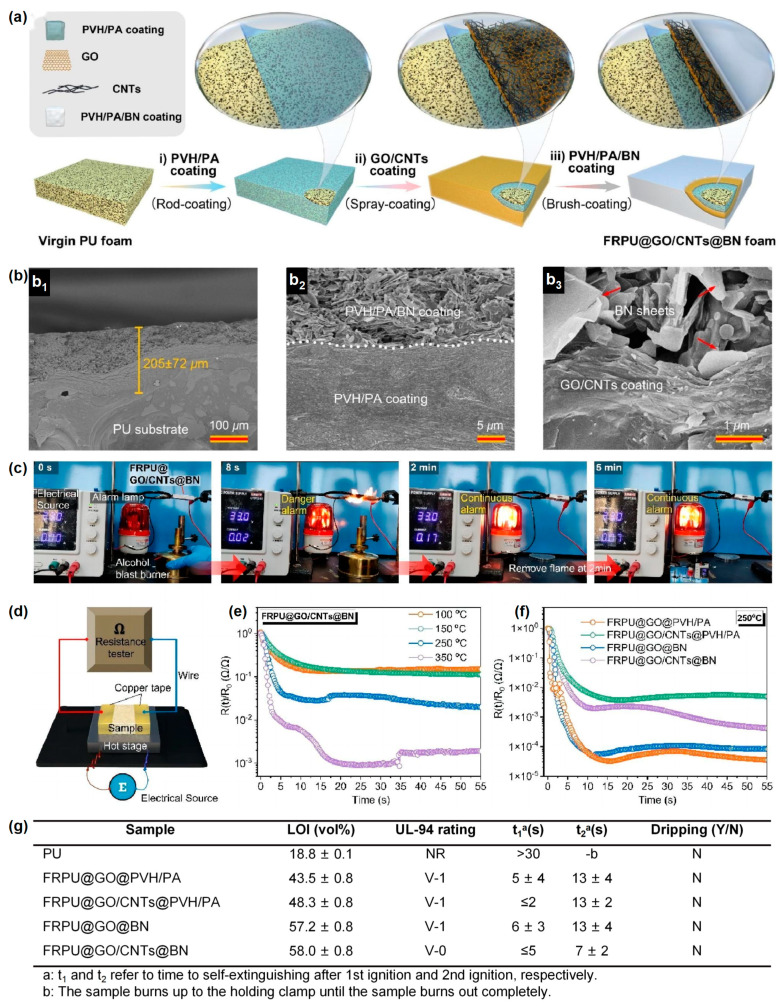
(**a**) Schematic diagram of the preparation of FRPU@GO/CNTs@BN foam. (**b**) SEM photographs of the FRPU@GO/CNTs@BN cross-section morphology at various magnifications. (**c**) Flame detection of FRPU@GO/CNTs@BN. (**d**) The apparatus for sensing resistance variation with temperature. (**e**) The FRPU@GO/CNTs@BN electrical resistance varies in real-time as a function of temperature. (**f**) Coated PU foams’ electrical resistance variations over at 250 °C. (**g**) LOI and UL-94 test results of PU and coated PU foams [74].

Phytic acid (PA), flame-retardant copolymer (PVH), GO, carbon nanotubes (CNTs), and BN are used as building blocks in a three-layer, sandwich-like flame-retardant coating of RPUF, as described by Ma et al. in their study [74]. Figure 18a illustrates the preparation of the FRPU@GO/CNTs@BN foam. The SEM image of the cross-sectional morphology of FRPU@GO/CNTs@BN at different magnifications is shown in Figure 18b. Figure 18c demonstrates that the flame detection method of FRPU@GO/CNTs@BN activates a hazard warning within 8 s and can continue to do so even when the burner is turned off. Figure 18d shows the resistance change detection device at different temperatures. As shown in Figure 18e, the test temperature has a direct effect on how the resistance changes the composite coating. The rate of resistivity change increases with temperature. The GO coating shows a greater change in resistivity than the GO/CNT coatings because the appearance of the CNTs only increases the initial conductivity of the coating and does not respond to temperature changes (Figure 18f). The resulting PU foam exhibits a rapid fire alarm response of about 8 s, even at high temperatures. It continues to warn of fire even when exposed to flames. 

### 4.4. Electromagnetic Shielding

The rapid development of electrical technology has made people’s lives more comfortable. Meanwhile, electromagnetic pollution has become a serious problem, affecting people’s health and the functionality of other equipment. The development of effective electromagnetic interference (EMI) protection materials is a practical method to reduce the propagation of electromagnetic waves [75,76,77].

**Figure 19:**
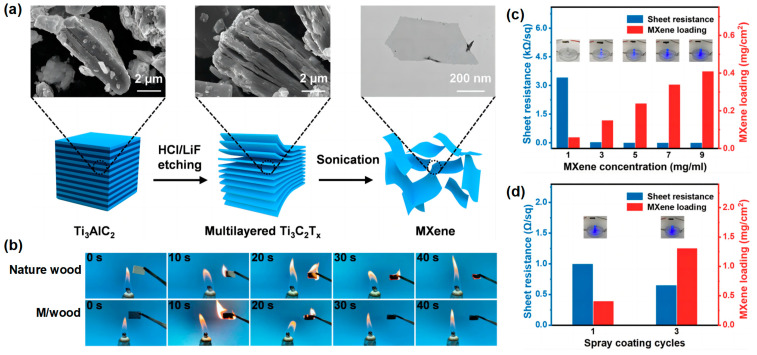
(**a**) Diagram of the preparation of the MXene sheets and the corresponding SEM images; (**b**) video screenshot of the burning process of natural wood and M/wood at various times. (**c**) The sheet resistivity of wood and MXene loading at various MXene concentrations. (**d**) The sheet resistivity of wood and the cycles of spray coating at various MXene concentrations; the M/wood electrical contact for LED lighting bulbs are seen in the picture [73].

Wei et al. [73] developed a simple and practical approach to produce highly conductive, flame-retardant wood with excellent EMI shielding properties. This coating can also be applied to RPUF. The SEM images and fabrication route of MXene are shown in Figure 19a. Figure 19b shows the digital images of the combustion process of natural wood and M/wood at different times, respectively. The presence of MXene can improve the flame resistance of wood. The effectiveness of the EMI shielding is significantly influenced by the conductivity. The conductivity of M/wood is investigated at different MXene concentrations and spray cycles. Natural wood acts as an insulator. As the MXene concentration increases, so does the sheet resistance. (i.e., the conductivity increases rapidly) (Figure 19c). The change in the conductivity of M/wood is also reflected in the change in brightness of the light-emitting diode illuminated by M/wood. The conductivity is positively correlated with the concentration of MXene, which helps in the preparation of a conductive M/wood preparation for practical use. In addition, after three spraying cycles of the MXene solution (9 mg/mL), a reduced M/wood sheet resistance (0.65/sq) is obtained (Figure 19d). This is because there are more free electrons to facilitate the overall charge transport properties and create a compact conductive network.

## 5. Summary and Outlook

Both the bulk and the surface flame-retardant strategies are effective at improving the flame-retardant properties of RPUF. While the bulk flame-retardant strategy reduces the mechanical property of the matrix to some extent, on the side, the surface flame-retardant strategy requires a greater coating thickness which is needed due to the close-cell structure of RPUF, so the combination of bulk flame retardant and surface flame retardant would compensate for the lack of both.

Whether it is a non-expandable coating or an expandable coating, some flame retardant is required to improve the flame-retardant performance. EG and APP/PER/MEL are relatively excellent choices, but more effective flame retardants also need to be designed and synthesized, among which phosphorus flame retardants cannot be ignored. In addition, renewable biomass resources are attracting increasing attention due to depleting oil reserves and increased environmental awareness. Cellulose has been shown to be effective as a flame retardant and other biomass materials need to be explored for coating applications.

Inspired by volcanic lava and tree frogs, flame retardancy and surface adhesion have been effectively improved. The invention of new RPUF flame-retardant coatings could also be inspired by nature.

The durability of SFR/RPUF depends on many factors. In surface finishing, poor adhesion between the coating and the substrate surface also reduce the durability of the flame retardant, and the interfacial compatibility problem also affects the overall performance of the materials. The interfacial adhesion performance and hydrophobicity mentioned in this paper need to be further addressed. The wide application range of RPUF also brings special requirements for its performance in different scenarios, such as fire warning and electromagnetic shielding, and the coating could be equipped with other functions. The simultaneous application of multifunctional coatings remains a major challenge.

## Data Availability

Not applicable.
